# The Angiotensin-Converting Enzyme Inhibitory State Promotes the Transformation of Non-Small Cell Lung Cancer Blood Supply Pattern Toward Vasculogenic Mimicry Formation

**DOI:** 10.3389/fonc.2021.663671

**Published:** 2021-06-16

**Authors:** Kandi Xu, Huize Han, Yexin Luo, Hong Ye, Hongxia Lin, Lei Ni

**Affiliations:** ^1^ Department of Respiration and Critical Care Diseases, Ruijin Hospital, School of Medicine, Shanghai Jiao Tong University, Shanghai, China; ^2^ Institute of Respiratory Diseases, School of Medicine, Shanghai Jiao Tong University, Shanghai, China; ^3^ Respiratory and Critical Care Center, Shanghai East Hospital, Tongji University, Shanghai, China; ^4^ First Clinical Medical College, Anhui Medical University, Anhui, China; ^5^ School of Foreign Studies, Anhui University, Anhui, China

**Keywords:** renin-angiotensin system, angiotensin-converting enzyme 2, vasculogenic mimicry, VE-cadherin, nodal, Notch4, non-small cell lung cancer

## Abstract

Tumor microenvironment plays an important role in tumor proliferation, metastasis, and angiogenesis. Local RAS is a key factor to tumor proliferation and metastasis in NSCLC microenvironment, but its role on angiogenesis and VM formation remains unclear. Although overwhelming majority of previous studies suggested that VM is well established in aggressive tumor and facilitates tumor growth and metastasis, we put forward different views from another angle. We proved that status of tumor blood supply patterns, including VM channels and endothelial vessels, can dynamically exchange with each other along with local RAS fluctuations in microenvironment. Quantitatively, ACE2/ACEI promotes VM formation *via* Nodal/Notch4 activation; while structurally, ACE2/ACEI leads to a strong and solid structure of VM *via* inhibition of VE-cadherin internalization. These changes induced by ACE2/ACEI relate to relatively low metastasis rate and comforting prognoses of NSCLC patients.

## Introduction

Lung cancer (LC), the most common cancer worldwide, due to its highly invasive and metastatic potential, is also the predominant cause of cancer-related death, of which non-small cell lung cancer (NSCLC) accounts for approximately 80% (1, 2). Tumor microenvironment plays an important role in tumor proliferation and metastasis. Hypoxia and angiogenesis, two of the most significant tumor environmental factors, are related to increased distant metastasis and poor survival in various tumor types (3, 4).

Due to tumor heterogeneity, an intricate tumor angiogenic pattern, considered to contain, among other things, vasculogenesis, sprouting angiogenesis, vessel co-option, vascular intussusception, vasculogenic mimicry, is becoming increasingly appealing currently (5). The existence of vasculogenic mimicry (VM), a novel blood perfusion way different from blood vessels, is found to be well established in aggressive tumor especially within an oxygen deficient environment, such as neuroblastoma, melanoma, and NSCLC (6–8). Cancer stem cells (CSCs), a subgroup of malignant tumor cells which express both endothelial and tumor markers, enable themselves to mimic vascular endothelial cells to participate in the formation of VM due to its plasticity. Comprised of cancer-derived endothelial-like cells in malignancies, VM has recently been recognized as a resistance mechanism of anti-angiogenesis inhibitor as it facilitates tumor growth and metastasis in anoxic environment. As biomarkers of VM, VE-cadherin and EphA2 co-express at cell junction of VM, and the interaction of which mediates VM like a switch. Moreover, classic signal pathway (PI3K/AKT and MAPK) and HIF-1*α* can motivate cancer cell growth, invasion, angiogenesis, and the generation of VM as well in NSCLC (9–11). There is another notion which doubts VM might be a degenerative trace of tumor vessels (12), but the structure and attribute of VM with different generation modes have not been compared.

The systemic renin–angiotensin system (RAS) controls the cardiovascular system, and the local RAS is a key factor to tumor proliferation and metastasis in tumor microenvironment. The expression of angiotensin II (Ang II), part of RAS, is increased locally in microenvironment and related to tumor growth, angiogenesis, and prognosis (13). However, the biological effect of Ang II on tumor can be reversed by angiotensin-converting enzyme inhibitors (ACEIs), angiotensin II type 1 receptor blockers (ARBs), or angiotensin-converting enzyme 2 (ACE2). Previously, we proved that the level of ACE2 in NSCLC tissues is reduced. Functionally, ACE2 can suppress tumor proliferation, invasion, angiogenesis, and metastasis by downregulating the expression of Ang II and vascular endothelial growth factor a (VEGFa) (14–16). Nevertheless, the role of ACE2 in VM and the underlying mechanisms are not clear.

In the present study, we explored the alternate pattern of angiogenesis in ACE2-overexpression model. We confirmed the overexpression of ACE2 promotes solid VM formation which is induced by Nodal/Notch4 and VE-cadherin in NSCLC.

## Materials and Methods

### Patient Samples and Tissue Microarray

All clinical investigations were approved by Ruijin Hospital Ethics Committee and conducted according to the Declaration of Helsinki. Eighty three NSCLC patients with detailed clinical and pathological information received surgery and follow-up (5 years after surgery) in Ruijin Hospital from 2013 to 2016. Patients who died within 1 month after surgery and who were lost during the follow-up were not enrolled in present study. In order to reduce the risk of bias due to confounding factors, we restricted sample by including only certain patients that have similar values of potential confounding variables (age, gender, race, BMI, comorbidity, *etc.*), and in the following univariate analysis, the reported association would not be confounded by other factors. All pathological patterns of removed tumor tissues used for TMA construction were double confirmed by the Pathology Department of Ruijin Hospital and Shanghai Outdo Biotech Co, Ltd (China). Two 1-mm cores were punctured in each tissue sample to create TMA, and the score of each sample was calculated as the mean score of two cores.

### Cell Culture

Human non-small cell lung cancer cell line A549 (Shanghai Institute of Cells, China) and NCI-H1650 (Cell Bank of the Type Culture Collection of the Chinese Academy of Sciences, China) were grown in Dulbecco’s modified Eagle’s medium supplemented with 10% fetal bovine serum (all from Gibco BRL, USA), 1% penicillin/streptomycin and 1% Glutamax at 37°C with 5% CO_2_.

### Plasmid Construction

Human ACE2 cDNA (5′ CGATCTTAATTAAATGCAGATGGCGGACGC-3′, 5′-TCAGTGGCGCGCCCTATTTGGAAAGTTTGCTTATAACTCTG-3′) was synthesized and cloned by polymerase chain reaction (PCR), then ligated into plenti6.3-MCS-IRES-EGFP (Yingrun Biotechnologies Inc., China, **Figure 3A**) between Pac1 and Asc1 restriction sites to construct the overexpression vector pLenti6.3-ACE2 in 293T cells.

The pLenti6.3-ACE2 plasmid was confirmed by PCR and sequencing.

### Overexpression of ACE2 in A549 Cells

Lenti-ACE2-EGFP and Lenti-EGFP, harvested from the supernatants, were added into A549 cells with polybrene (8 µg/ml) and incubated for 24 h to obtain ACE2 overexpressing A549 cells (A549-ACE2-OE cells) and ACE2 negative control cells (A549-NC cells), (MOI = 20) respectively.

Transfection efficiency, fluorescence of reporter gene EGFP in both cell lines, were observed using a fluorescence microscope. Western blot was carried out to examine the ACE2 protein expression level in A549-ACE2-OE cells and A549-ACE2-NC cells.

### Overexpression Plasmid and Transient Transfection in NCI-H1650

ACE2 overexpression plasmid (pENTER-ACE2) and a control vector were designed and constructed by Vigene Biosciences Inc. (Jinan, China). NCI-H1650 cells, 6 × 105, were transfected with 1.5 μg pENTER-ACE2 using Lipo6000™ transfection reagent (C0526, Beyotime, Shanghai, China) according to the manufacturer’s instructions. Protein samples were extracted 72 h after transfection.

### Western Blot

Equal amounts of protein samples were extracted from cells and separated by sodium dodecyl sulfate polyacrylamide gel electrophoresis, transferred onto a polyvinylidene fluoride membrane (Millipore, USA), blocked with 5% fat-free milk, then incubated with antibodies against ACE2 (1:3,000, ab108252, Abcam, US), VE-cadherin (1:1,000, ab33168, Abcam), EphA2 (1:1,000, 6997s, CST, USA), AKT (1:1,000, 4691, CST), p-AKT (1:2,000, 4060, CST), p38 (1:2,000, ab7952, Abcam), p-p38 (1:1,000, 4511, CST), Nodal (1:2,000, ab55676, Abcam), Notch4 (1:2,000, ab184742, Abcam), and Actin (1:2,000, ab8226, Abcam) overnight at 4°C, and the corresponding secondary antibodies (1:5,000, Thermo Fisher, USA) for 1 h at room temperature (RT). The blots were examined by an enhanced chemiluminescence detection kit (Millipore). Actin was used as a loading control, and all experiments were repeated three times independently. Expression quantification of proteins was performed using ImageJ software (National Institutes of Health, USA).

### Real-Time Quantitative Reverse Transcription-Based Polymerase Chain Reaction

Total RNA was extracted using the TRIzol reagent and subjected to reverse transcription and qPT-PCR according to the manufacturer’s instructions (all from Takara, Japan). Relative expression level of ACE2, VE-cadherin, and EphA2 was assessed by the comparative quantification in triplicate. ACTB was an internal control.

The primer sequences used for qRT-PCR are listed in **Table 1**.

**Table 1:** The primer sequences of genes used in qRT-PCR.

Genes	Primer sequences (5′−3′)	Products
**ACTB**	F: GGCACTCTTCCAGCCTTCC	255 bp
	R: GAGCCGCCGATCCACAC	
**ACE2**	F: TGGCTACAGAGGATCAGGAGT	2418 bp
	R: GAACTTGGGTTGGGCGCTATT	
**VE-cadherin**	F: CATCGGTTGTTCAATGCGTC	118 bp
	R: GGTACATGACAGAGGCGTGG	
**EphA2**	F: CCGTATGGCAAAGGGTGG	236 bp
	R: TCGGCATAGTAGAGGTTGAAAGT	

### Three-Dimensional Culture

Wells of 24-well plate coated with a mixture of Matrix and Type I Collagen (both from BD Biosciences, the Netherlands, 1:1, 250 µl per well) were laid for 1 h at 37°C. 60 × 10^4^ A549-ACE2-OE cells, or A549-NC cells with or without Captopril (concentration: 1, 5, and 10 nM/L, HY-B0368, MedChemExpress, New Jersey, USA) were seeded onto each well. Images were captured using a Leica DMi8 microscope, and net structure was analyzed by ImageJ.

### Immunohistochemistry and Scoring System

Protein expression was detected by IHC in fixed, embedded paraffin sections of allograft tissues and TMAs. The sections were dewaxed, rehydrated, treated with antigen retrieval, hatched with primary antibodies of ACE2 (1:200), VE-cadherin (1:200), EphA2 (1:100), and CD34 (1:200, ab81289, Abcam) overnight at 4°C, individually exposed to secondary antibody (SA1020, BOSTER, USA) 1 h at RT, and colored with DAB peroxidase substrate. After counterstaining with hematoxylin, sections were recorded with a Leica-SP8.

For TMAs, a semi-quantitative scoring manner was utilized in a high-power field (HPF: 40× magnification) of each core visually by two pathologists blinded to patient information and the aim of this study. Staining intensity of cells was graded as follows: 0 = undetectable, 1 = weak, 2 = moderate, 3 = high. Percentage of stained cells was graded as follows: 0: 0–1%; 1: 2–25%; 2: 26–50%; 3: 51–75%; 4: 76–100%. The overall scoring points were calculated as the product of score of staining intensity of cells and percentage of stained cells.

For allograft tissues, Image-Pro Plus 6.0 soft was run to obtain the mean optical density of four HPFs in each section.

### Periodic Acid-Schiff Stain

A PAS staining kit (395B, Sigma, USA) was applied after CD34 IHC (CD34/PAS double staining) or alone to take count of endothelial micro vessel (MV) and tumor-derived VM of TMAs or four HPFs in each tissue section. Briefly, the sections were exposed to 1% sodium periodate (10 min) and Schiff’s buffer (10 min) at 37°C in turn.

MV is considered to be lined by CD34^+^/PAS^−^ endothelial cells, while VM is regarded as closely arranged by CD34^−^/PAS^+^ tumor cells whose nuclei are large and hyperchromatic within blood cells or free cancer cells.

### Immunofluorescent Staining

Cells, 4 × 10^4^, were plated on glass slides and cultured overnight. Then cells were fixed with 4% paraformaldehyde (15 min) and permeabilized with 0.5% Triton-100 for 10 min. Four percent BSA was used to block non-specific protein–protein interactions (1 h). The cells were treated with antibody against human HIF1-a (ab92498, Abcam) overnight followed by anti-rabbit Alexa Fluor 488 (ab150077, Abcam) and Alexa Fluor 594 (R37117, Thermo Fisher) secondary antibodies. After counterstaining with DAPI, cells were recorded, and the mean optical density was researched by Image J.

### Animal Experiments

C57BL/6 mice of 5–6 weeks were purchased from Bethesda (USA) and randomly divided into three groups (A549-NC; A549-ACE2-OE; A549 + ACEI). The corresponding cells (5 × 10^6^) were injected (s.c.) into the mice flanks, and a common ACEI drug Captopril (0.3 mg/kg) was used (i.g.). Tumor length and width were measured by a caliper every 4 days, and the volume was calculated (tumor volume = (length * width^2^)/2). Twenty days later or when the tumor volumes reached 1,000 mm^3^, the mice were euthanized and tumors were isolated. The removed tumors were weighed and underwent subsequent tests. Laparotomies were performed to clear the distant metastasis of vital organs. All animal operations were carried out in accordance with institution guidelines of Ruijin Hospital.

### Statistical Analysis

All data analyses were performed with SPSS 23.0 (IBM, USA) and GraphPad Prism 6.0 (GraphPad Software Inc., USA). If not mentioned, the data are shown as mean ± SD. The groupings of patients are all based on the median of variable data sets, respectively. Differences between groups were analyzed by two-tailed Student’s t-test (for quantitative data) and Chi-square test (for categorical data). OS of patients was calculated by Kaplan–Meier survival analysis, and differences were analyzed by log-rank (Mantel–Cox) test. Pearson’s test was used for correlation analysis. P ≤0.05 was considered statistically significant.

## Results

### High ACE2 Expression Was Linked to Increased VM and Better Prognosis in NSCLC

**Figure 1:**
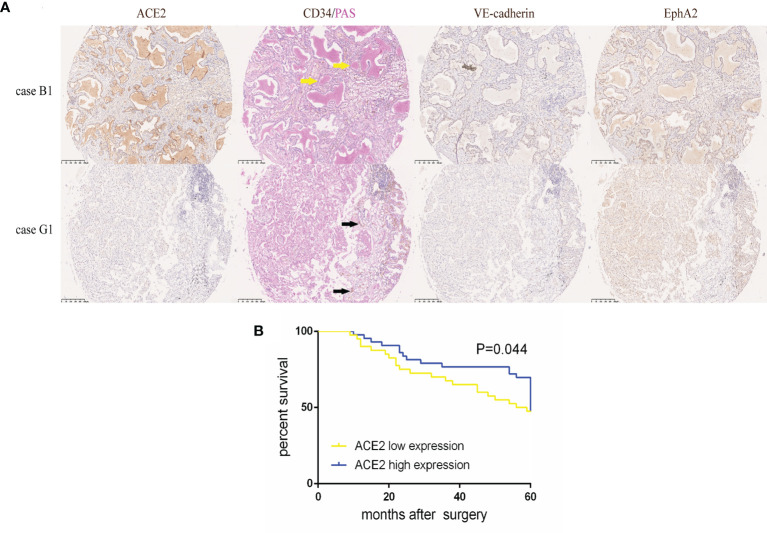
High ACE2 expression was linked to increased VM and better prognosis in NSCLC. **(A)** Typical image of ACE2, VE-cadherin, EphA2 protein expression and CD34/PAS double staining in TMA tissues. Case B1 had massive CD34^−^/PAS^+^ VM (yellow arrows) lined by ACE2, VE-cadherin and EphA2 high expressing tumor cells. Case G1 had abundant CD34^+^/PAS^−^ MVs (black arrows) with ACE2, VE-cadherin and EphA2 low expressing tumor cells. **(B)** Kaplan-Meier analysis of OS in NSCLC patients with ACE2 low or high expression. P = 0.044.

To accurately study the association between ACE2 status and disease progression in NSCLC, 83 patients who had accepted radical excisions were dichotomized into either ACE2 low (≤1) or high (>1) expression group with a cut-off value, the median ACE2 score of excised tumors. Whereafter we did a comparative analysis of clinicopathological data of the two groups (**Table 2**). Patients with low ACE2 expression were mostly female (62.5 *vs* 37.2%, P = 0.021) or smokers (75.0 *vs* 53.5%, P = 0.042), concealed more advanced vascularization (39.38 ± 39.33 *vs* 23.03 ± 27.43, P = 0.032), and owned a reduced 5-year survival (47.5 *vs* 71.4%, P = 0.027); by comparison, more patients among the ACE2 high expression group retained VM generation (44.2 *vs* 20.0%, P = 0.019), VE-cadherin (VE-cadherin score > 0, 53.5 *vs* 30.0%, P = 0.030), EphA2 (EphA2 score ≥ 8, 72.1 *vs* 50.0%, P = 0.039) high expression, escaped recurrence or metastasis 5 years after surgery (46.5 *vs* 25.0%, P = 0.042). In TMA, VM frequently occurred in NSCLC tissues with ACE2 high level in the form of regular and integrated small pipes or large blood lakes (**Figure 1A**), and the majority of ACE2^+^/VE-cadherin^+^/EphA2^+^ cells were assembled into them; howbeit immature and discontinuous CD34^+^ (regarded as an endothelial marker) MVs were easily caught in ACE2 low expressing tissues. Univariate survival analysis also affirmed that compared with patients in ACE2 high expressing group, patients with ACE2 low expressing status had a poorer 5-year survival (P = 0.044, **Figure 1B**).

**Table 2:** The relativity between ACE2 expression and clinicopathological characteristics in NSCLC patients.

Factors		ACE2 low expression patients(n = 40, %)	ACE2 high expression patients(n = 43, %)	P value
**Sex**	Male	15 (37.5)	27 (62.8)	0.021
	Female	25 (62.5)	16 (37.2)	
**Age (years)**	<65	25 (62.5)	26 (60.5)	0.849
	≥65	15 (37.5)	17 (39.5)	
**Smoking**	No	10 (25.0)	20 (46.5)	0.042
	Yes	30 (75.0)	23 (53.5)	
**Primary tumor** **size (diameter)**	≤3	25 (62.5)	25 (58.1)	0.685
	>3	15 (37.5)	18 (41.9)	
**Clinical stage**	I	20 (50.0)	20 (46.5)	0.936
	II	4 (10.0)	5 (11.6)	
	III	16 (40.0)	18 (41.9)	
**Lymph node** **status**	N0	21 (52.5)	24 (55.8)	0.696
	N1	3 (7.5)	5 (11.6)	
	N2	16 (40.0)	14 (32.6)	
**CEA**	Negative	22 (57.9)	27 (64.3)	0.558
	Positive	16 (42.1)	15 (35.7)	
**CYFRA21-1**	Negative	26 (66.7)	30 (73.2)	0.526
	Positive	13 (33.3)	11 (26.8)	
**NSE**	Negative	31 (79.5)	37 (86.0)	0.430
	Positive	8 (20.5)	6 (14.0)	
**5-year prognosis**	Survival	19 (47.5)	30 (71.4)	0.027
	Death	21 (52.5)	12 (28.6)	
**5-year recurrence or** **metastasis**	Negative	10 (25.0)	20 (46.5)	0.042
	Positive	30 (75.0)	23 (53.5)	
**MV**		39.38 ± 39.33	23.03 ± 27.43	0.032
**VM**	Negative	32 (80.0)	24 (55.8)	0.019
	Positive	8 (20.0)	19 (44.2)	
**VE-cadherin**	Negative	28 (70.0)	20 (46.5)	0.030
	Positive	12 (30.0)	23 (53.5)	
**EphA2**	Low expression	20 (50.0)	12 (27.9)	0.039
	High expression	20 (50.0)	31 (72.1)	

### ACE2-Induced Better Outcome in NSCLC Patients Might Be Attributed to Less Vessels and More VM Formation

**Figure 2:**
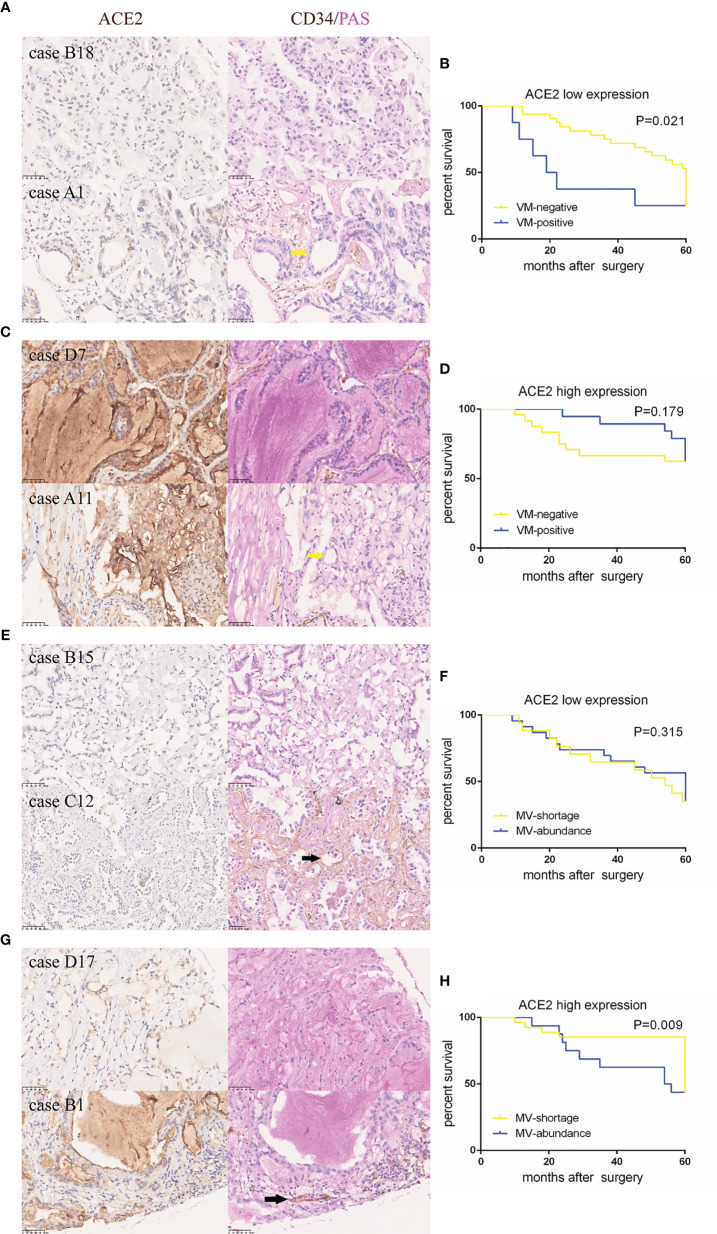
ACE2-induced better outcome in NSCLC patients might be attributed to less vessels and more VM formation. **(A, C, E, G)** Typical tissue images of each group stained with ACE2 or CD34/PAS. Yellow arrows: CD34^−^/PAS^+^ VMs; black arrows: CD34^+^/PAS^−^ MV. **(B, D, F, H)** Kaplan–Meier analysis of OS in each group.

To make further efforts on the relation of the transformed pattern of blood supply triggered by ACE2 with prognosis, patients were further divided into two groups according to the presence of VM and the number of MV (cut-off value: 10), respectively. Noticeably, in ACE2 low expressing group, positive VM presence was related to shorter periods of NSCLC patients than negative VM presence (P = 0.021, **Figures 2A, B**); but with respect to patients with ACE2 high level, there was no significant difference in survival between two groups (P = 0.179, **Figures 2C, D**). On the other hand, patients with shortage of MV possessed better prognoses than those with abundance of MV in ACE2 high level group (P = 0.009, **Figures 2G, H**), but this kind of advantage no longer existed in ACE2 low level group (P = 0.315, **Figures 2E, F**). In summary, the alternate pattern of angiogenesis promoted by ACE2 plays a decisive role in NSCLC prognosis.

### Human ACE2 Was Stably Overexpressed in A549-ACE2-OE Cells

**Figure 3:**
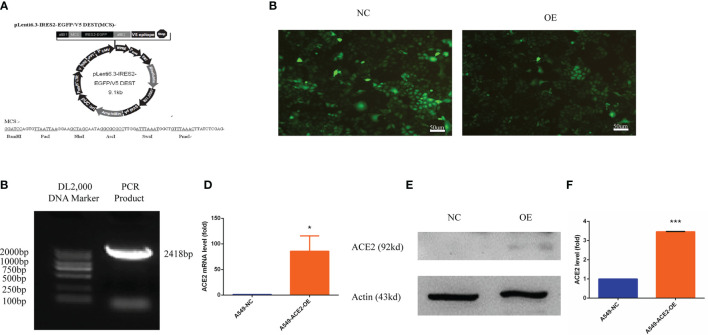
Human ACE2 was stably overexpressed in A549-ACE2-OE cells. **(A)** Schematic representation of pLenti6.3-MCS/V5 DEST. **(B)** pLenti6.3-ACE2 expression vector was detected by PCR. **(C)** Fluorescence of EGFP in A549-ACE2-OE cells (left) and parental cells (right) was determined by fluorescence microscopy. **(D)** RT-PCR experiment of ACE2 mRNA level in A549-ACE2-OE cells and control cells, Mean ± SD, n = 3, *p < 0.05. **(E)** Western blot analysis of ACE2 expression level in A549-ACE2-OE cells and parental cells. **(F)** Quantification of ACE2 expression level in A549-ACE2-OE cells and parental cells. Mean ± SD, n = 3, ***p < 0.001.

To intensively assess the effect of ACE2 on VM formation of NSCLC, an ACE2 overexpressing A549 cell model was established. pLenti6.3-ACE2 expression vector was successfully constructed using pLenti6.3-MCS/V5 DEST (**Figure 3A**) and was detected by PCR and sequencing. The PCR product post restriction enzyme digestion shared the same number of bases (2,418 bp) with ACE2 gene (**Figure 3B**). A549 cells were transfected with pLenti6.3-ACE2 expression vector (A549-ACE2-OE) or empty vector as a negative control (A549-NC), then fluorescence of EGFP in both cell lines was observed to determine the transfection efficiency and photographically recorded (**Figure 3C**). Next, the persistent overexpression of ACE2 by lentivirus was demonstrated by RT-PCR and Western blot analysis 72 h later (**Figures 3D, E**), and there was a more than three-fold increase of ACE2 protein expression in A549-ACE2-OE cells shown in **Figure 3F**, compared with negative control. All these data above suggested an ACE2 overexpressing A549 cell line was successfully constructed.

We also transiently transfected NCI-H1650 with pENTER-ACE2 and determined the transfection efficiency by western blotting (**Supplement 1A, B**).

### Tube Formation Ability of A549 Cells Was Improved With ACE inhibitory State

**Figure 4:**
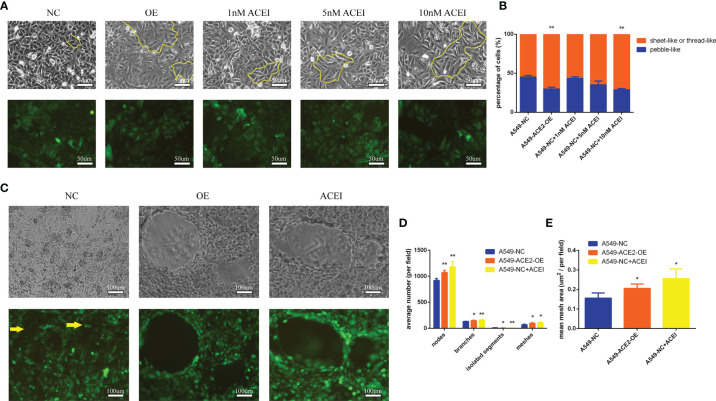
Tube formation ability of A549 cells was improved with ACE inhibitory state. **(A)** Morphologies of a panel of A549-ACE2-OE cells, A549-NC cells, and A549-NC cells treated with ACEI (1, 5, and 10 nM/L) were shown as sheet-like and thread-like cell types in 2D culture, which were outlined partly with yellow lines. Representative images were shown above. Upper: white light; lower: fluorescence. **(B)** Quantification of sheet-like or thread-like cells and pebble-like cells in three groups, representatively, Mean ± SD, n = 3, **p < 0.01. **(C)** Images of both above cell lines grown in 3D Matrix gel. A 10 nM/L ACEI dilution was performed. Representative images were shown above. Yellow arrows point out the free cancer cells escaping from tube wall and isolated segments. Upper: white light; lower: fluorescence. **(D, E)** Images of 3D culture were applied to determine average number of nodes, branches, isolated segments, meshes and mean mesh area in those groups, per field, Mean ± SD, n = 3, **p < 0.01, *p < 0.05. ns, no significance.

Surprisingly, we found a significant difference of morphologies between A549-ACE2-OE cells and paired cells following a 72-h culture in conventional 2D culture plates: different percentages of sheet-like or thread-like cell types were shown in A549-ACE2-OE cells and parental cells (**Figure 4A**). A549-ACE2-OE cells, growing in a more dispersed state, provided many more elongated spouting cells than pebble-like cells, compared with A549-NC cells (**P < 0.01, **Figure 4B**), which means the overexpression of ACE2 might harbor a trans-differentiation of endothelial features in A549 cells. Next, the capability of tube formation of both above cell lines was examined by 3D Matrix gel culture and images were digitally captured 72 h later (**Figure 4C**). Notably, more nodes (**P < 0.01), branches (*P < 0.05), and meshes (*P < 0.05) were produced in tubes formed by A549-ACE2-OE cells, with an increased mean mesh size (*P < 0.05), while more isolated segments were found in negative control (*P < 0.05, **Figures 4D, E**). Thus, A549-ACE2-OE cells were competent to shape larger, more massive and more substantial tubes, yet tubes carried out by A549-NC cells were few, scattered, and fragile.

We went a step further to the pharmacological action of ACEI on VM formation *in vitro*. Hereby a series of ACEI dilution was added into A549 cell culture system, and an adequate concentration (10 nM/L, **P < 0.01) was found. ACEI has an analogous effect on VM formation to ACE2 overexpression in a dose-dependent manner in conventional 2D culture and 3D culture.

### VM Formation Was Increased and Vasculature Was Lessened Due to Inhibition of RAS *In Vivo*

**Figure 5:**
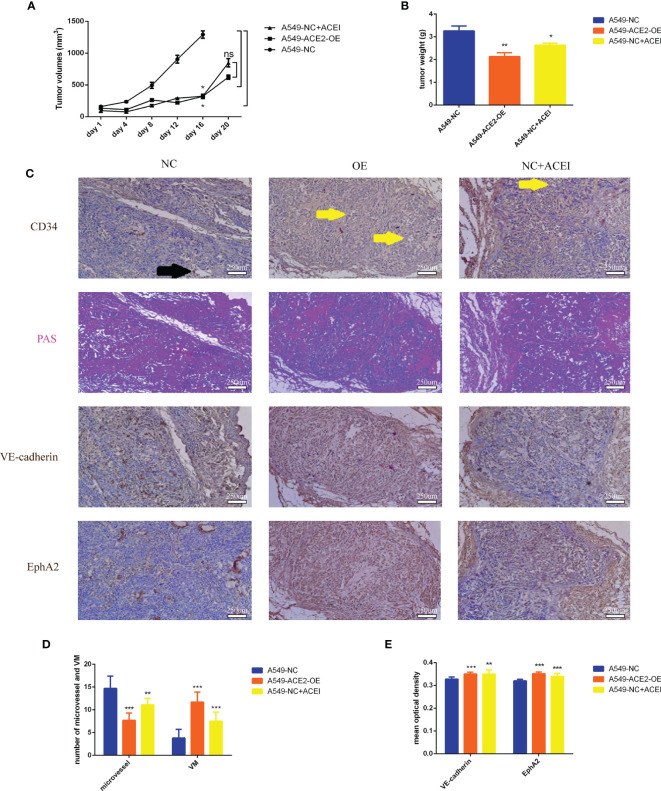
VM formation was increased, and vasculature was lessened due to inhibition of RAS *in vivo*. **(A)** Growth curve of allograft tumors of A549-ACE2-OE cells, A549-NC cells with or without ACEI treatment, Mean ± SD, n = 3, *p < 0.05. ns, no significance. **(B)** Weight of resected tumors, Mean ± SD, n = 3, **p < 0.01, *p < 0.05. **(C)** Continuous sections of allograft tumor tissues stained with PAS, CD34, VE-cadherin, or EphA2 immunohistochemical stain. Black arrow points out a typical MV (CD34^+^/PAS^−^); yellow arrows point out typical VM (CD34^−^/PAS^+^). **(D)** Quantification of MV and VM in different groups, Mean ± SD, n = 3, per field, ***p < 0.001, **p < 0.01. **(E)** Quantification of VE-cadherin and EphA2 mean optical density in three groups, Mean ± SD, n = 3, ***p < 0.001, **p < 0.01.

To better prove changes of angiogenesis pattern resulting from local RAS in tumor microenvironment, mice were injected with A549-ACE2-OE cells or A549-NC cells, administered with ACEI. Tumor growth was abated in A549-ACE2-OE group and ACEI group (*P < 0.05, *P < 0.05, **Figure 5A**), respectively, compared with that in A549-NC group. Xenografts were also lightened (**P < 0.01, *P < 0.05, **Figure 5B**). All mice (3/3) from the A549-NC group had distant multiple organ metastasis, whereas in A549-ACE2-OE group and ACEI group there was one (1/3) and zero (0/3). Consequently, tumor loads were alleviated by both ACE2 overexpression and ACEI treatment.

Furthermore, consecutive sections of allograft tumor tissues, stained with PAS, CD34, VE-cadherin, or EphA2 (**Figure 5C**), were scanned to count the number of MV (CD34^+^/PAS^−^) and VM (CD34^+^/PAS^−^). We validated allayed micro vessels (***P < 0.001, **P < 0.01, **Figure 5D**) and multiplied VM (***P < 0.001, ***P < 0.001, **Figure 5E**) should be ascribed to ACE2 augmentation and ACE inhibition.

### VE-Cadherin and EphA2 Expression Was Upregulated in A549 Cells and NSCLC Tissues With Impaired Local RAS Status

**Figure 6:**
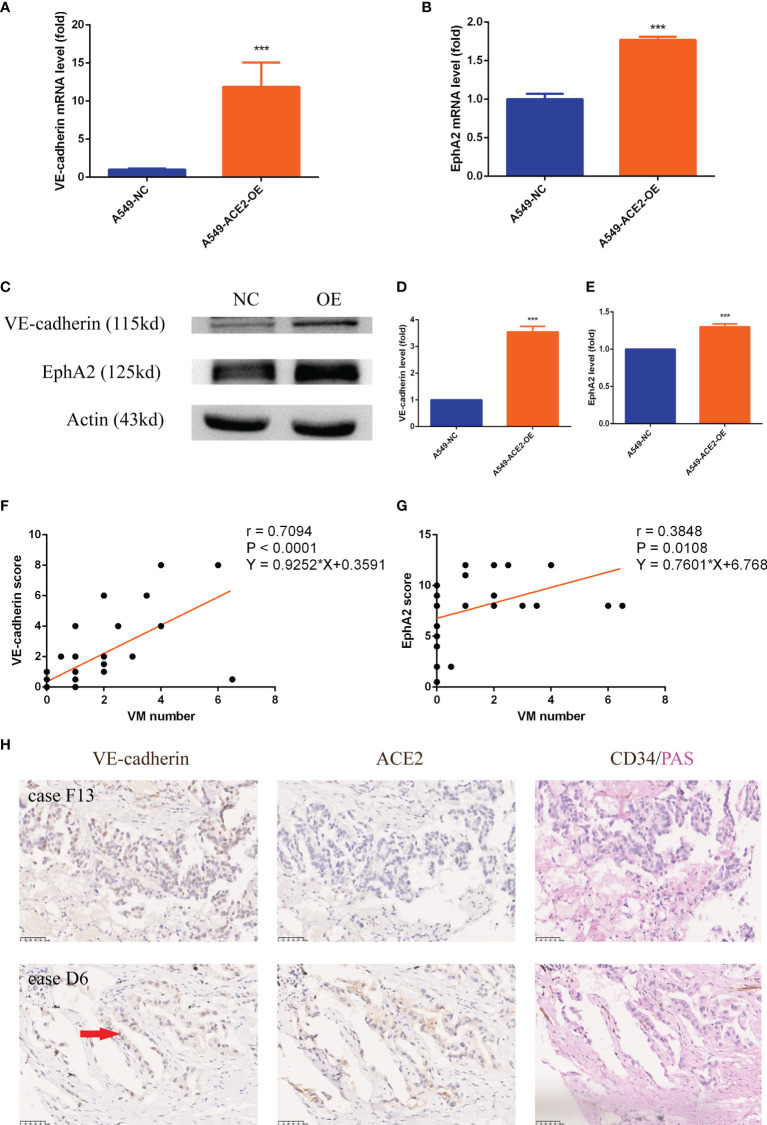
VE-cadherin and EphA2 expression was upregulated in A549 cells and NSCLC tissues with impaired local RAS status. **(A, B)** RT-PCR experiment of VE-cadherin and EphA2 mRNA level in A549-ACE2-OE cells and control cells, Mean ± SD, n = 3, ***p < 0.001. **(C–E)** Western blot analysis and quantification of VE-cadherin and EphA2 expression level in A549-ACE2-OE cells and control cells, Mean ± SD, n = 3, ***p < 0.001. **(F, G)** Linear regressions of VM number and VE-cadherin (P < 0.0001) or EphA2 (P = 0.0108) score in TMA. **(H)** Typical tissue images of both groups stained with VE-cadherin, ACE2 or CD34/PAS. Case F13 with ACE2 low status was provided with rambling VM covered by tumor cells which only expressed VE-cadherin in nuclei; case D6 with ACE2 high status had ordered VM lined by tumor cells expressing VE-cadherin in both nuclei and cytomembranes. Red arrow: VE-cadherin membrane expression.

The accepted crucial VM molecular markers VE-cadherin and EphA2 expression were detected in our ACE2 overexpressing A549 cell model after 3D Matrigel culture. We announced that, by contrast, the elevated ACE2 expression led to approximately 10 times enhancement of VE-cadherin mRNA expression by RT-PCR experiment (***P < 0.001, **Figure 6A**), accompanied by marked upregulation of EphA2 mRNA (***P < 0.001, **Figure 6B**). Accordingly, there were similar trends in VE-cadherin (***P < 0.001, **Figures 6C, D**) and EphA2 (***P < 0.001, **Figures 6C, E**) protein expression level confirmed by Western blot analysis. Similar results were obtained upon transient transfection of NCI-H1650 (**Supplement 2A**).

VE-cadherin (***P < 0.001, **P < 0.01) and EphA2 (***P < 0.001, ***P < 0.001) expression was also heightened in ACE2 high expressing and ACEI disposed allograft tumor tissues (**Figures 5C, E**). In ACE2 high expressing patient cohort, we found that VM number positively corelated with both VE-cadherin (r = 0.7094, P < 0.0001, **Figure 6F**) and EphA2 (r = 0.3848, P = 0.0108, **Figure 6G**) expression at their protein level. Tissues with VE-cadherin^+^/VM^+^ in TMA were reviewed, then 2/7 of patients in ACE2 low expressing group and 8/18 of patients in ACE2 high expressing group were found to be expressing VE-cadherin on membrane of tumor cells (**Figure 6H**).

These results proved that VE-cadherin and EphA2 are worthy of molecular markers in ACE2-triggered VM.

### PI3K/AKT, p38MAPK, HIF1-a Were Inactivated and Nodal/Notch4 Pathway Was Activated in A549-ACE2-OE Cell Model

Apart from adhesion factors, the changes of vascularity-related classic signal pathways, hypoxia and embryonic/stem cell signaling caused by ACE2 overexpression were unraveled in turn.

**Figure 7:**
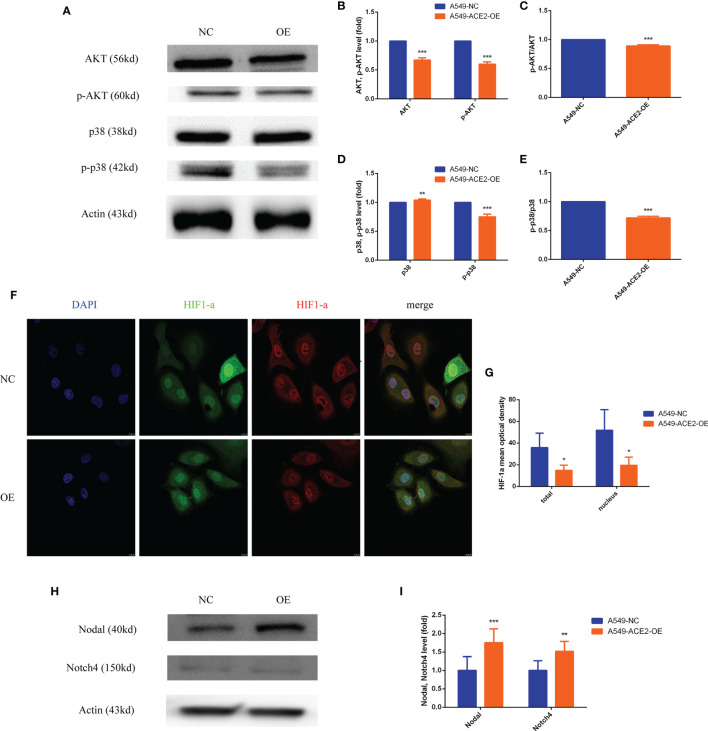
PI3K/AKT, p38MAPK, and HIF1-a were inactivated, and Nodal/Notch4 pathway was activated in A549-ACE2-OE cell model. **(A–E)** Western blot analysis and quantification of AKT, p-AKT, p38, and p-p38 expression level in A549-ACE2-OE cells and negative control, Mean ± SD, n = 3, ***p < 0.001, **p < 0.01. **(F, G)** Immunoflurescence assay and quantification of HIF1-a mean optical density in A549-ACE2-OE cells and negative control, Mean ± SD, n = 4, *p < 0.05. **(H, I)** Western blot analysis and quantification of Nodal and Notch4 expression level in A549-ACE2-OE cells and negative control, Mean ± SD, n = 3, ***p < 0.001, **p < 0.01.

Both AKT and p-AKT expression levels were abated relatively (***P < 0.001, ***P < 0.001, **Figures 7A, B**), and what is more, AKT activation by phosphorylation was restrained in A549-ACE2-OE cells compared with paired cells (***P < 0.001, **Figure 7C**). Consistent results were discovered in p38 and p-p38 (**P < 0.01, ***P < 0.001, **Figures 7A, D**, ***P < 0.001, **Figure 7E**).

As for HIF1-a, immunofluorescence affirmed that overexpressing ACE2 remarkably reduced total HIF1-a expression (*P < 0.05) and nuclear HIF1-a accumulation (*P < 0.05) compared with control (**Figures 7F, G**).

Contrarily, compared with that in negative control, Nodal (***P < 0.001) and Notch4 (**P < 0.01) expression levels were correspondingly increased in A549-ACE2-OE cell model (**Figures 7H, I**). Similar results were obtained upon transient transfection of NCI-H1650 (**Supplement 3A, B**).

## Discussion

The status of tumor blood supply patterns, including VM channels, mosaic blood vessels, and endothelial vessels, can dynamically exchange with each other along with fluctuations in microenvironment (11). The dynamic balance of local RAS system in tumor is proved to be of great concern to tumor homeostasis. Bound to angiotensin type 1 receptor (AT1), Ang II, with elevated expression in tumor microenvironment, is able to facilitate tumor progression and angiogenesis and results in poor prognosis (13). As part of the negative feedback mechanism in RAS, ACE2 converts Ang II to Ang-(1-7), a peptide with vasodilator and anti-proliferative properties. Besides, ACEI, stopping the transition of Ang I to Ang II catalyzed by ACE, still has a controversial impact on incidence and mortality of cancer (17–19). Previously, description of local RAS regulation among NSCLC blood supply patterns was confined to endothelial vessels, but VM was rarely involved.

VM closely relates to tumor progression, metastasis, and poor survival rate and is generally considered to be a mark emblem of serious neoplastic conditions (5). In the current study, ACE2 protein had a heterogeneous expression in NSCLC tissues, and high ACE2 expression might be protective and might be linked to a better prognosis in NSCLC. During ACE suppression (including ACE2 high status and ACEI-treated condition), NSCLC patients or animal model gained better outcomes, and in addition, this benefit might be achieved by means of both reduced MV and increased VM formation, which seems to contradict general knowledge. Earlier reports hinted VM could also be a consequent of vessel normalization, which usually occurs after anti-VEGF (bevacizumab) and capecitabine therapy (12, 20, 21). Regular and integrated VM in ACE2 high level group should be stronger than immature and discontinuous MV in the low group in barrier function. Therefore, naive vessels might be replaced by solid VM in ACE2 high status to ensure low permeability and guarantee the chemotherapy drugs will arrive at intratumoral designated location.

Apart from vessel normalization, whether there is an ACE2-induced spontaneous VM formation needs to be made clear. An ACE2-OE cell line was used alone to exclude interference from endothelial cells, then quantitative and structural differences were shown. Due to more isolated segments in A549-NC cell production, cancer cells outlining the tubes (**Figure 4G**, yellow arrows) were more readily released into matrix gel, which could reveal the origin of circulating tumor cells on the mechanism and the potential function of VM of prognostic prediction in NSCLC. ACE2 overexpressing endowed NSCLC cells with endothelial phenotype and VM building capacity, but ACE2-induced VM was not as fragile as the general one, which might profit from tight intercellular connections (22).

We elaborated ACE2/ACEI-related VM also had characteristic VE-cadherin and EphA2 expression. Endothelial cells’ barrier function is mediated in part by homotypic binding of transmembrane adherent junction proteins such as VE-cadherin. Post-translational VE-cadherin modifications trigger VE-cadherin internalization and increase vascular permeability, which can modulate tumor cell extravasation (23–25). VE-cadherin seemed more likely to appear on membranes of tumor cells instead of nuclei at the ACE2/ACEI-related VM border, which explained solid frame and low probability of metastasis.

Both ACE2/ACEI-related VM and angiogenesis function on tubular fluid-conduction and share vascularity-related classic signal pathways as well as hypoxia cell signaling. Since ACE2/ACEI regulated MV and VM differently, candidate pathways were checked and Nodal/Notch4 was found to be activated in ACE2/ACEI-related VM. Nodal/Notch belongs to the superfamily of the transforming growth factor *β* (TGF-*β*) (11). The crosstalk of Notch and Nodal participates in embryonic stem cell maintenance and VM formation in melanoma, breast cancer cells (26). However, Nodal is not typically expressed in normal adult tissues including mature vascular endothelial cells. Activated Nodal/Notch4 may mediate enhanced plasticity and VM formation of ACE2/ACEI-treated NSCLC cells.

More attractive, high ACE2 level might prevent females and smokers from NSCLC. Our sample size had limitation, and no multivariable analysis was performed. Patients with high grade clinical stage had no surgical indications and were excluded from this study, which might lead to the irrelevance of ACE2 level and clinical stage. In this research, insufficient cell lines or animals were applied. Because of incomplete medical history, ACEI impact on local RAS and VM was not explored. These defects will be improved in future studies.

Here, we try to portray a macro picture of dynamic change between vascularization and VM formation within aberrant local RAS. In NSCLC, the generation of VM was promoted by ACE2/ACEI, but the generation of MV was inhibited; ACE2/ACEI led to a strong and solid structure of VM; in ACE2 high level group, patients with VM had better prognoses. VM function is determined by structure, and it seems, not only quantity, but also structure and quality are part of the measure of VM function. High ACE2 level might indicate a relatively comforting prognosis, which suggests an up-to-date direction of NSCLC treatment; but patients with low ACE2 status need to be closely monitored after surgery.

High ACE2 expression improves VM formation quantitatively and structurally, which is conducive to prognosis of NSCLC patients.

## Data Availability Statement

The original contributions presented in the study are included in the article/**Supplementary Material**. Further inquiries can be directed to the corresponding author.

## Ethics Statement

The studies involving human participants were reviewed and approved by Ethics Committee of Shanghai Jiao Tong University School of Medicine Affiliated Ruijin Hospital. The patients/participants provided their written informed consent to participate in this study. The animal study was reviewed and approved by Ethics Committee of Shanghai Jiao Tong University School of Medicine Affiliated Ruijin Hospital.

## Author Contributions

KX and LN designed this study. KX, HH, and HL performed the experiments. YL performed the data analysis. HY and KX wrote this manuscript. All authors agree to be accountable for the content of the work. All authors contributed to the article and approved the submitted version.

## Funding

Our study is supported by the Shanghai Key Discipline for Respiratory Diseases (2017ZZ02014) and the Science and Technology Commission of Shanghai Municipality (15ZR1426200).

## Conflict of Interest

The authors declare that the research was conducted in the absence of any commercial or financial relationships that could be construed as a potential conflict of interest.
