# Supplementary material for: The Angiotensin-Converting Enzyme Inhibitory State Promotes the Transformation of Non-Small Cell Lung Cancer Blood Supply Pattern Toward Vasculogenic Mimicry Formation
Source: Front Oncol. 2021 Jun 16;11:663671. doi: 10.3389/fonc.2021.663671 (PMC8242235; doi:10.3389/fonc.2021.663671)
Supplement: Supplementary file 1 [file DataSheet_1.docx]

Supplementary Material


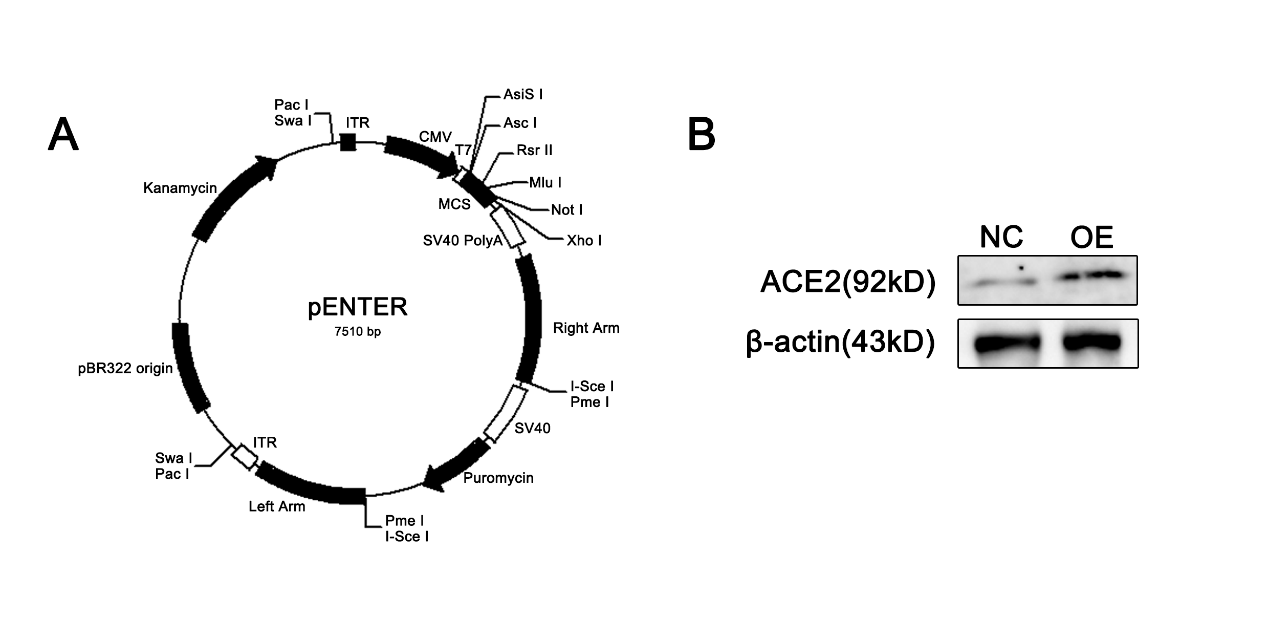


**Supplement 1**. Human ACE2 was transient overexpressed in NCI-H1650 cells. **(A)** Schematic representation of pENTER. **(B)** Western blot analysis of ACE2 expression level in NCI-H1650 cells transiently transfected with pENTER-ACE2. Cells transfected with the parental plasmid were used as the control.


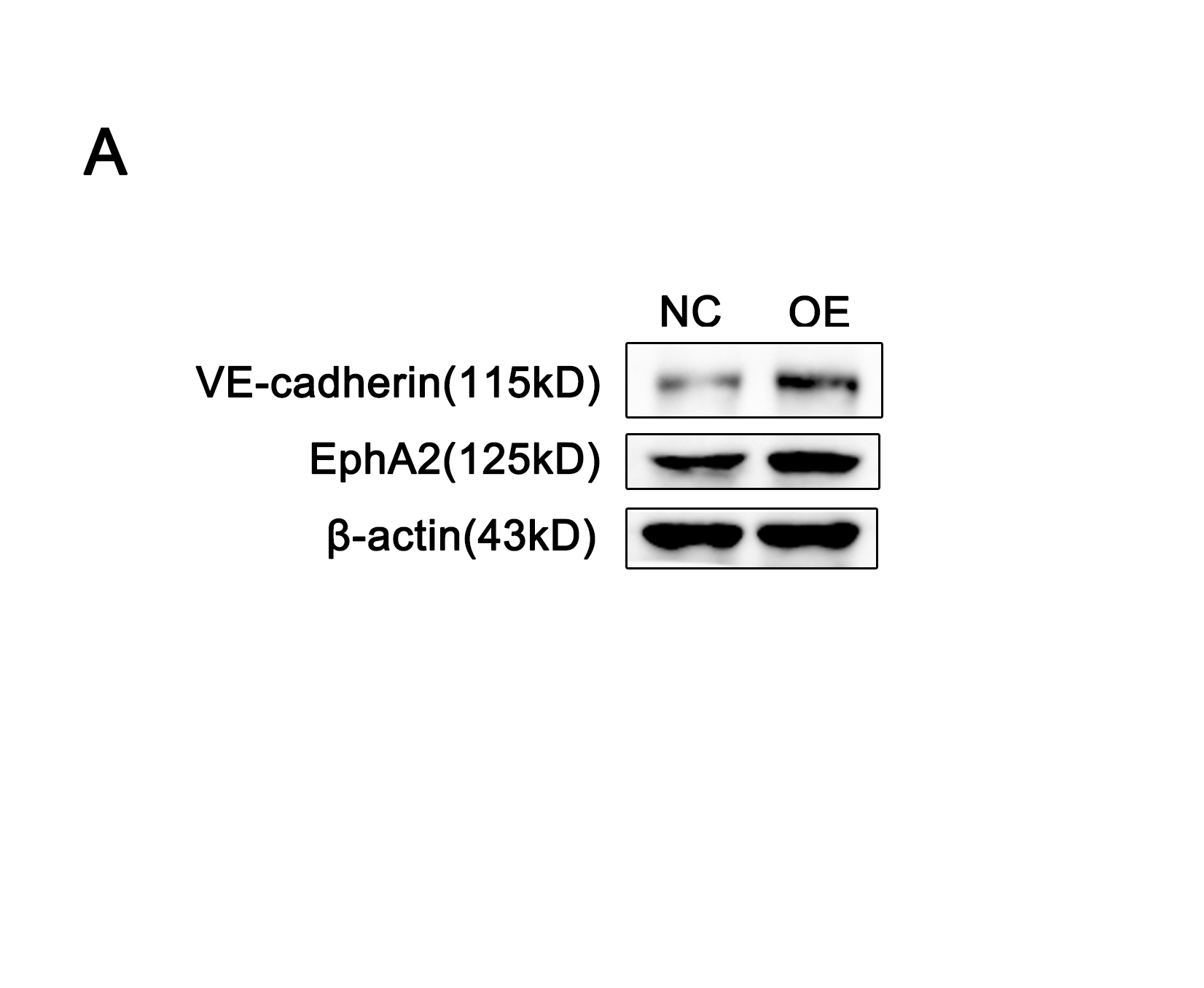


**Supplement 2**. VE-cadherin and EphA2 expression was upregulated in NCI-H1650 cells transiently transfected with pENTER-ACE2. **(A)** Western blot analysis of VE-cadherin and EphA2 expression level in NCI-H1650 cells transiently transfected with pENTER-ACE2. Cells transfected with the parental plasmid were used as the control.


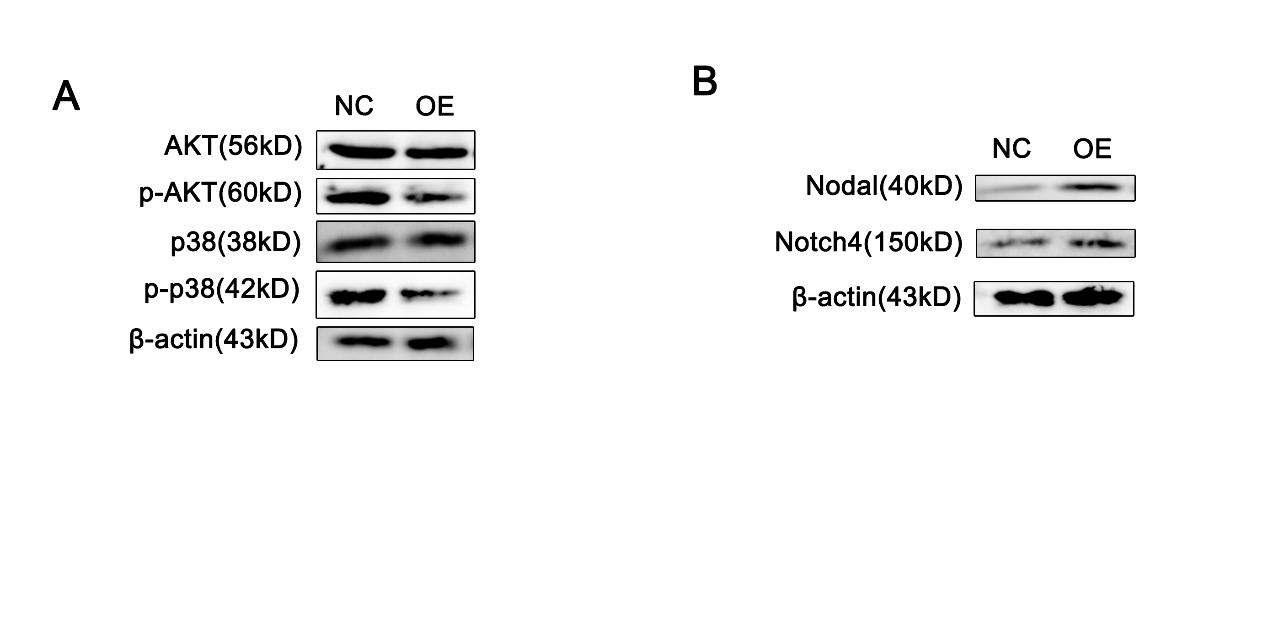


**Supplement 3**. PI3K/AKT, p38MAPK, HIF1-a were inactivated and Nodal/Notch4 pathway was activated in NCI-H1650 cells transiently transfected with pENTER-ACE2. **(A)** Western blot analysis of AKT, p-AKT, p38 and p-p38 expression level in NCI-H1650 cells transiently transfected with pENTER-ACE2. Cells transfected with the parental plasmid were used as the control. **(B)** Western blot analysis and of Nodal and Notch4 expression level in NCI-H1650 cells transiently transfected with pENTER-ACE2. Cells transfected with the parental plasmid were used as the control.
